# Novel CD20 monoclonal antibodies for lymphoma therapy

**DOI:** 10.1186/1756-8722-5-64

**Published:** 2012-10-11

**Authors:** Shundong Cang, Nikhil Mukhi, Kemeng Wang, Delong Liu

**Affiliations:** 1 Department of Oncology, People’s Hospital, Henan Province, China; 2 Department of Medicine, New York Medical College and Westchester Medical Center, Valhalla, NY, 10595, USA

## Abstract

Rituximab (RTX), a monoclonal antibody (mAb) against CD20, has been widely used for lymphoma therapy. RTX in combination with cyclophosphamide /doxorubicin /vincristine /prednisone (R-CHOP) remains the standard frontline regimen for diffuse large B-cell lymphoma. However, suboptimal response and /or resistance to rituximab have remained a challenge in the therapy of B-cell non-Hodgkin’s lymphoma (NHL). Novel agents are under active clinical trials. This review will summarize the latest development in new mAbs against CD20, which include second-generation mAbs, ofatumumab, veltuzumab (IMMU-106), ocrelizumab (PRO70769), and third-generation mAbs, AME-133v (ocaratuzumab), PRO131921 and GA101 (obinutumumab).

## Background

Rituximab (RTX), a monoclonal antibody (mAb) against CD20, has been widely used for lymphoma therapy [1-4]. RTX in combination with cyclophosphamide /doxorubicin /vincristine /prednisone (R-CHOP) remains the standard frontline regimen for diffuse large B-cell lymphoma (DLBCL) [5,6]. However, suboptimal response and /or resistance to rituximab have remained a challenge in the therapy of B-cell non-Hodgkin’s lymphoma (NHL). Novel agents are under active clinical trials. This review will summarize the latest development in new mAbs against CD20.

### Rituximab, the first-generation CD20 monoclonal antibody

**Figure 1:**
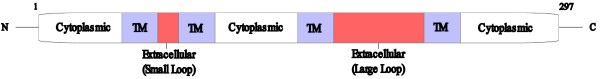
**The structure of CD20 molecule. Human CD20 is encoded by the gene MS4A1 gene located on chromosome 11q12.2.** CD20 molecule is a 297 amino acid phosphoprotein with four transmembrane domains.

**Figure 2:**
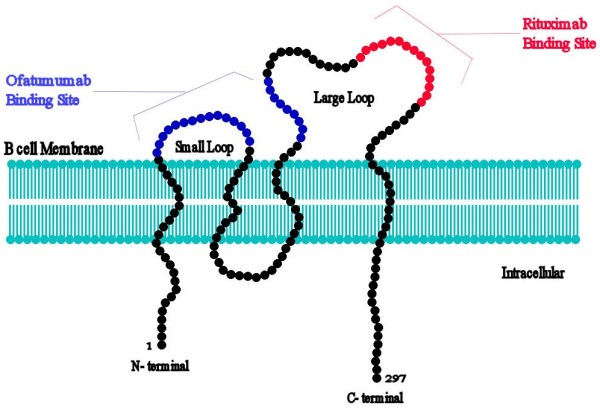
**The molecular configuration of CD20 molecule.** CD20 is a tetra-transmembrane protein that essentially remains on the membrane of B cells without dissociation or internalization upon antibody binding. The binding sites of CD20 monoclonal antibodies, rituximab and ofatumumab, are indicated.

CD20 is the first B-cell specific antigen defined by the monoclonal antibody tositumomab [7,8]. Human CD20 is encoded by the gene MS4A1 gene located on chromosome 11q12.2 [9]. CD20 molecule is a 297 amino acid phosphoprotein with four transmembrane domains (Figure 1). It plays a critical role in B-cell development. CD20 has been a superb biomarker for immunotherapies targeting B-cell derived diseases [10]. It is known to function through binding to Src family tyrosine kinases, such as Lyn, Fyn, and Lck, and believed to be involved as a result in phosphorylation cascade of intracellular proteins. It is a tetra-transmembrane protein that essentially remains on the membrane of B cells without dissociation or internalization upon antibody binding (Figure 2) [11]. RTX, the first generation CD20 mAb, can induce complement-dependent cytotoxicity (CDC) and antibody-dependent cellular cytotoxicity (ADCC), leading to its clinical activity against lymphoma cells [12]. CDC represents the primary mechanism for cell-killing by RTX. However, some lymphoid cells ( *i.e*. 10% of CLL cells) were resistant to CDC because of lower levels of complement activation or decreased cytotoxicity of activated complements. In addition, RTX can lead to apoptosis of B cells upon binding to CD20 and therefore can directly inhibit cell growth [13]. Recently, a novel mechanism of cell killing by mAbs was reported to involve reactive oxygen species mediated through NADPH [14]. 

RTX has been widely used for lymphoma therapy [1-4,15-17]. R-CHOP chemotherapy remains the standard regimen for newly diagnosed DLBCL [3,5,18]. CD20 mAbs conjugated to nuclear isotopes have also been approved for lymphoma therapy [18-22]. RTX has also been studied in new combination regimens, particularly for relapsed and refractory lymphomas [23-31].

To increase antitumor activity and Fc binding affinity for the low-affinity FcγRIIIa receptor (CD16) on immune effector cells, new generations of anti-CD20 monoclonal antibodies (mAbs) are being developed [7,8,11].

### Second-generation anti-CD20 mAbs

The second-generation anti-CD20 mAbs include ofatumumab, veltuzumab, and ocrelizumab. These are humanized to reduce immunogenicity.

Ofatumumab (OFA) is a fully human type I anti-CD20 IgG1kappa mAb [15,32-37]. Ofatumumab binds to both the small and large extracellular loops (ECL) of CD20 molecule, and is more effective than rituximab at killing target cells. It has been shown to be more potent than RTX against both RTX- sensitive and resistant cells [38]. Its activity against RTX-resistant cells and the potent CDC effect are believed to be due to the proximal epitope of the small loop of CD20 molecule and the high capacity for C1q activation.

OFA (arzerra) has been approved for treatment of relapsed or refractory CLL who have failed fludarabine and alemtuzumab (FA-ref) [39]. OFA is given IV weekly on a fixed dose, 300 mg for dose 1, and 2000 mg weekly x 7 in subsequent doses. This is followed by every four weeks for 4 more doses [36]. In FA-ref CLL patients, the response rate was 58%. OFA was also studied in 116 patients with RTX-refractory follicular lymphoma (FL) [40]. These patients received 8 weekly infusions (dose 1, 300 mg and doses 2–8, 500 or 1000 mg). The median age was 61 years, and the median number of prior therapies was 4. The overall response rate (ORR) was 13% and 10% for the 500-mg and 1000-mg arms, respectively (Table 1). The median PFS was 5.8 months. Infections, rash, urticaria, fatigue, and pruritus were the most common adverse events. Severe adverse events included neutropenia, anemia, and thrombocytopenia in a subset of patients. 

**Table 1:** Second generation CD20 monoclonal antibodies

**Monoclonal Ab**	**Ig type**	**Patients**	**Regimen**	**Overall response rate(%)**	**Adverse events**	**Reference**
**Ofatumumab (OFA)**	IgG1 Kappa	116	Ofatumumab (Refractory FL) 500 mg 1000mg	13% 10%	Infections, rash, urticaria, pruritis, neutropenia, anemia, thrombocytopenia	[40]
		59	O-CHOP (untreated FL) 500 mg 1000 mg	90% 100%	Leucopenia, neutropenia	[41]
		61	O-FC (Frontline therapy for CLL) 500 mg 1000 mg	77% 73%	Neutropenia, thrombocytopenia, anemia, infection	[35]
**Veltuzumab**	IgG1	82	Veltuzumab (Relapsed/refractory B cell NHL) 80 to 750 mg/m^2^	44%(FL) 83%(MZL) 43%(DLBL)	Fatigue, pruritis, asthenia, fever, dyspnea, headache, infection	[42]
**Ocrelizumab**	IgG1	47	Ocelizumab (Relapsed/Refractory FL) 750mg/m^2^	38%	Infusion related reaction, nasopharyngitis, asthenia, lymphopenia, infection	[43]

Abbreviations: DLBCL: diffuse large B cell lymphoma; FL: follicular lymphoma; MZL: marginal zone lymphoma; NHL: non-Hodgkin lymphoma.

OFA is being explored in combination with other agents in various B-cell neoplasms. An international, phase II trial was conducted to assess two doses of ofatumumab combined with CHOP (O-CHOP) for frontline treatment for follicular lymphoma [41]. The study randomized 59 patients to receive either OFA 500 mg (n = 29) or 1000 mg (n = 30) on day 1, with CHOP on day 3 every 3 weeks for six cycles. ORR was 90% for the 500-mg group and 100% for the 1000-mg group (Table 1). CR/CRu was 62%. The most common severe adverse events were leucopenia (29%) and neutropenia (22%). O-CHOP was reported to be safe and effective as frontline therapy for FL patients.

Another international randomized phase II trial was done using OFA in combination with fludarabine 25 mg/m^2^ and cyclophosphamide 250 mg/m^2^ on days 2–4 in course 1 and on days 1–3 for courses 2–6 [35]. This was repeated every 4 weeks for 6 courses. Two OFA dose levels, 500 mg (n = 31) or 1000 mg (n = 30), were given to patients with untreated CLL after randomization. The first OFA dose was 300 mg for both cohorts. The median age of the study pts was 56 years; 17p deletion was seen in 13% of patients. CR rate was 32% for the 500-mg and 50% for the 1000-mg cohort. ORR was 77% and 73%, respectively. Beta_2_-microglobulin and the number of O-FC courses given correlated significantly with CR, ORR, PFS, according to univariate analyses (P < .05). Neutropenia (48%), thrombocytopenia (15%), anemia (13%), and infection (8%) were found to be the most common severe adverse events. The study concluded that O-FC is active and safe in treatment-naive CLL patients, including high-risk patients with 17p deletion.

Veltuzumab (IMMU-106, hA20) has more potent binding avidities and a stronger effect on CDC than rituximab [44]. Veltuzumab is a humanized, type I anti-CD20 IgG1 mAb, engineered recombinantly with complementarity-determining regions (CDRs) identical to rituximab, except for a single amino acid change, Asp101 instead of Asn101, in the CDR3 of the variable heavy chain [10,44]. This modification results in significantly slower off-rates and increased CDC cytotoxicity in three human lymphoma cell lines. Clinically, veltuzumab has been studied in over 150 patients with lymphomas and autoimmune diseases. In a multicenter phase I/II dose-finding study of veltuzumab in relapsed/refractory B-cell non-Hodgkin's lymphoma (NHL), eighty-two patients (median age, 64 years; 79% stage III/IV, one to nine prior treatments) received infusions of 80–750 mg/m^2^ once-weekly for four doses, with no grade 3 to 4 drug-related adverse events other than infusion-related reactions [42]. Complete response (CR) was seen in 27% of FL patients despite failure of two to five prior rituximab-containing regimens. ORR for FL, MZL, and DLBCL was 44%, 83%, and 43%, respectively (table 1). The median duration of responses was 19.7 months. B cell depletion was seen at all dose levels studied. The depletion occurred after the first infusion. The veltuzumab serum half-lives were similar after the fourth infusion, and mean antibody serum levels were higher than the values generally considered important for anti-CD20 therapy (*i.e*., 25 mg/ml). Subcutaneous injections of low doses (80–320 mg) were also studied and have also proved to be safe and pharmacologically active [45].

Ocrelizumab (PRO70769) is another type I second generation humanized mAb that differs from rituximab at several amino acid positions within the CDRs of the light chain and heavy chain variable regions. With enhanced efficacy toward lymphoid malignancies and increased binding affinity for the low-affinity variants of the FcγRIIIa receptor (CD16), this mAb has increased ADCC and lower CDC activity compared with rituximab. This mAb has been evaluated in a phase I/II study for safety and efficacy in patients with relapsed/refractory follicular lymphoma (FL) after failing prior RTX therapy [43]. Forty-seven patients were enrolled in three dose cohorts and received eight infusions every 3 weeks: cohort A, 200 mg/ m^2^ (n = 15); cohort B, 375 mg/ m^2^ (n = 16); cohort C, first dose 375 mg/ m^2^ with seven subsequent doses at 750 mg/ m^2^ (n = 16). The patients had a median age of 58 years and had received a median of 2 (range 1–6) prior regimens. The grade 3/4 toxicity occurred in 9% of patients. The most common toxicity was infusion-related reactions which occurred in 74% of the patients. The ORR was 38% regardless of affinity variants in the FcγRIIIa. The median follow-up was approximately 28 months, and the median PFS was 11.4 months.

### Third-generation anti-CD20 mAbs

The third-generation humanized CD-20 mAbs have an engineered Fc region to increase their binding affinity for the FcγRIIIa receptor. Three third-generation mAbs, AME-133v, PRO131921 and GA101, are undergoing active clinical development.

AME-133v (LY2469298, ocaratuzumab) is a type I, humanized IgG1 mAb. Its binding affinity to CD20 has a 13 to 20- fold increase with 5 to 7- fold higher avidity to the low-affinity (F/F and F/V) variants of FcγRIIIa receptor. These may have been the mechanisms to overcome the lower response rates and shorter duration of responses to rituximab. A phase I study was done with dose escalation in 10 Japanese patients with relapsed and /or refractory FL. It was administered by intravenous infusion at 100 or 375 mg/m^2^ weekly for 4 weeks. Nine patients were F-carriers while one was homozygous for valine (V/V) at position 158 of FcγRIIIa. There were no dose-limiting toxicities. Five (50%) of ten patients responded to ocaratuzumab treatment (three CR, one unconfirmed CR and one partial response). Ocaratuzumab was well tolerated and clinical activity was observed in FL patients pretreated with rituximab, mostly consisting of F-carriers [46]. Another phase I dose escalation study from US has also been reported in 23 relapsed FL patients. The dosages were well tolerated from 2 up to 375 mg/m^2^[47]. This highest dose was used in a phase II trial in 44 patients with relapsed FL following prior rituximab [48]. These patients with the low-affinity FcγRIIIa polymorphism (F-carriers) received 375 mg/m^2^ of ocaratuzumab weekly for 4 doses. The ORR was 36% and median progression free survival (PFS) reached 91 weeks (Table 2). In this analysis, there were 56 patients who received 100 and 375 mg/m^2^ of ocaratuzumab. Eight of these patients had a median of 2 prior rituximab treatments (range 1–6). Five of the 8 patients showed a longer PFS after ocaratuzumab administration, compared with last rituximab treatment. These 5 patients had the F/F low-affinity genotype of FcγRIIIa. Three of the patients had prolonged remission [48]. Ocaratuzumab will need to be compared to rituximab in randomized clinical trials to substantiate its potential clinical advantages. 

**Table 2:** Third Generation CD20 monoclonal antibodies

**Monoclonal Ab**	**Ig type**	**Patients**	**Regimen**	**Overall responserate**	**Adverse events**	**Reference**
**Obinutuzumab (GA-101)**	IgG1	21	Obinutuzumab (Refractory B cell NHL) 1600/800 mg 400/400 mg	60% 35%	Infusion related reaction, neutropenia, anemia, thrombocytopenia, tumor lysis syndrome	[49]
		28	G-CHOP (Relapsed or refractory FL) 1600/800 mg 400/400 mg	94%	Infusion related reaction, neutropenia, neuropathy, infection	[50]
		28	G-FC (Relapsed or refractory FL) 1600/800 mg 400/400 mg	93%	Infusion related reaction, neutropenia, rash, infection	[50]
**PRO131921**	IgG1	24	PRO131921 (Relapsed/refractory B cell NHL) 25 to 800 mg/m^2^	27%	Infusion related reaction, upper respiratory tract infection, neutropenia	[51]
**Ocaratuzumab (AME-133v)**	IgG1	56	Ocaratuzumab (Relapsed/Refractory FL) 100 mg/m^2^ 375mg/m^2^	36%	Infusion related reaction, nasopharyngitis, asthenia, lymphopenia, infection	[48]

PRO131921 is another humanized anti-CD20 mAb recombinantly engineered for enhanced CDC and ADCC activities over RTX [15,52,53]. In preclinical in vivo studies, it was shown to have better anti-tumor efficacy than rituximab. PRO131921 was given to patients (pts) with relapsed and /or refractory indolent lymphoma who failed rituximab-containing regimen [51]. This phase I study determined the maximum tolerated dose (MTD) and the pharmacokinetics (PK). PRO131921 was infused weekly for 4 weeks on days 1, 8, 15 and 22. The dose of the first infusion was half of subsequent infusions. Twenty-four pts were treated with PRO131921 at doses escalated from 25 mg/m^2^ to 800 mg/m^2^. A median of 2 (range 1-6) prior regimens were found in these pts. PRO131921 had no MTD in this study. The most common adverse events were infusion-related reactions, limited mostly to the first infusion. Dose limiting toxicity (DLT) included a significant infusion reaction and grade 3 joint pain and fatigue after 2 infusions. There was a correlation between drug exposure and tumor shrinkage (p=0.049) as well as clinical response (p=0.034). This suggested that drug clearance (e.g. by tumor in excess of drug) is proportional to clinical efficacy. Twenty two of the 24 pts were evaluable at the time of report in 2009. There were 6 partial response (PR 27%), 13 stable disease (SD), and 3 disease progression (PD). Half of 10 pts in the two highest dose cohorts responded. Further clinical trials are underway.

GA101 (RO5072759, obinutuzumab) is a fully humanized, type II, IgG1 mAb derived from humanization of the parental B-Ly1 mouse antibody and subsequent glycoengineering of Fc region [54-57]. GA101 binds CD20 through a totally different orientation than rituximab and over a larger epitope. It appears to have more potent activity through direct killing as well as NK-cell mediated ADCC effect. GA101 was shown to have activity in RTX-resistant cell lines [58,59].

In an open label multicenter, phase I/II study, GA101 was evaluated in patients with relapsed /refractory CD20+ NHL/CLL [60]. In the non-randomized dose-escalating phase I (3 + 3 design), 21 patients were treated with GA101 (50–2,000 mg) on days 1 and 8 of cycle 1, and day 1 in cycles 2–8 (21-day cycles). ORR was 56% (CR=31%, PR=25%). In the phase II part of the study, patients with indolent NHL were randomized to one of two doses: 1,600/800 mg (n = 22, ORR 60%), or 400/400 mg (n = 18, ORR 35%). The median follow-up time was 23.1 months. The median PFS for patients with FL was 11.8 months for the high-dose cohort, and 6.0 months for the low dose cohort (HR: 0.77 [95% CI 0.34–1.77). The most common adverse events (AE) were infusion-related reactions (400/400 mg: 72%; 1,600/800 mg: 73%). Responses were also seen among the 5 patients who were retreated with GA101. PK data from the studies have suggested that responding patients metabolize GA101 more slowly than non-responders [61].

Obinutuzumab (GA101) in combination with cyclophosphamide /doxorubicin /vincristine /prednisone (G-CHOP) or fludarabine /cyclophosphamide (G-FC) has been studied in patients with CD20+ relapsed/refractory FL in an open-label, multicenter, randomized Phase Ib study (BO21000). Response rates were 94% for G-CHOP and 93% for G-FC. Serum levels of GA101 from these pts receiving combination chemotherapies were measured by ELISA. GA101 PK data were reported [50]. The serum concentrations of GA101 corresponded to the GA101 dose level that pts received, regardless of chemotherapy arm.

GA101 represents the first third-generation type II glycoengineered CD20 mAb which entered randomized phase II/III clinical trials. GA101 was compared to RTX in a randomized phase II study in CLL/NHL patients [62]. In this study, a total of 175 pts (149 FL) were randomized to receive 4 weekly infusions of either GA101 (1000 mg, n=87) or rituximab (375 mg/m^2^, n=88). Maintenance treatment with GA101 or rituximab every 2 months for up to 2 years at the same dose was given to responders. ORR was the primary endpoint in the FL population. In this population, ORR for GA101 was 43.2 (32/74) *v* 38.7 (29/75) for rituximab. The CR/CRu rate was 10.8 in the GA101 arm *v* 6.7 for rituximab. Therefore, this first head to head trial of GA101 against RTX demonstrated higher ORR and similar adverse events. Phase III trials of GA101 in combination with chemotherapy are ongoing.

### Conclusions and future directions

Although RTX and newer mAbs against CD20 have revolutionized lymphoma therapy, a significant population of patients still succumbs to lymphomas. Novel agents with different mechanism of actions are being explored [63-76]. Bortezomib is an active agent for refractory mantle cell and other lymphomas [77-85]. Lenalidomide, an immunomodulatory agent, has been studied for lymphoma therapy [67,86]. mTOR inhibitors, everolimus and temsirolimus, are being studied for treatment of refractory and relapsed lymphomas [87-94]. New biomarkers, such as microRNAs, STATs and Tregs, appear to be useful for assisting lymphoma diagnosis and for developing new therapeutic agents [65,74,75,95-97]. Novel antibodies directed against lymphocyte-specific antigens, such as CD19 [98-101], CD22 [102-112], and CD30 [113-116], have shown promises for clinical applications. Combination regimens among these novel agents may provide further improvement on the outcome of lymphoma therapy.

## Competing interest

The authors have no relevant conflicts of interest.

## Authors’ contributions

All authors have contributed to data preparation, drafting and revising the manuscripts. All authors have read and approved the final manuscript.

## Author details

^1^Department of Oncology, People’s Hospital, Henan Province, China.^2^Department of Medicine, New York Medical College and Westchester Medical Center, Valhalla, NY 10595, USA.
